# Emerging Role of Epigenetic Modifiers in Breast Cancer Pathogenesis and Therapeutic Response

**DOI:** 10.3390/cancers15154005

**Published:** 2023-08-07

**Authors:** Richard Sean Lee, Kirti Sad, Dorelle V. Fawwal, Jennifer Marie Spangle

**Affiliations:** 1 Department of Radiation Oncology, Emory University School of Medicine, Atlanta, GA 30322, USA; richard.sean.lee@emory.edu (R.S.L.); kirti.sad@emory.edu (K.S.); dory.fawwal@emory.edu (D.V.F.); 2 Department of Biology, Emory College, Atlanta, GA 30322, USA; 3 Biochemistry, Cell & Developmental Biology Graduate Program, Emory University School of Medicine, Atlanta, GA 30311, USA

**Keywords:** breast cancer, epigenetics, HDAC, HAT, histone methylation, histone acetylation, chromatin modification

## Abstract

**Simple Summary:**

Worldwide, breast cancer is among the most frequently diagnosed cancers in women and the second leading cause of cancer-associated mortality in women. While tumor genetics contribute to therapies used to treat breast cancer and inform patient prognosis, modifications of histone proteins, which help organize the genetic material in cells into chromatin, can also influence therapeutic response and ultimately patient outcome. Here, we explore the role of enzymes that modify histone proteins in breast cancer. We discuss how these reversible, or “epigenetic”, changes to chromatin perturb the tumor environment and describe preclinical studies that provide a rationale for targeting chromatin-modifying enzymes in breast cancer. We build on existing preclinical data to connect epigenetic activity with changes in cells that support oncogenic growth and, when applicable, assess the current clinical status of treatments that target chromatin-modifying enzymes in breast cancer.

**Abstract:**

Breast cancer pathogenesis, treatment, and patient outcomes are shaped by tumor-intrinsic genomic alterations that divide breast tumors into molecular subtypes. These molecular subtypes often dictate viable therapeutic interventions and, ultimately, patient outcomes. However, heterogeneity in therapeutic response may be a result of underlying epigenetic features that may further stratify breast cancer patient outcomes. In this review, we examine non-genetic mechanisms that drive functional changes to chromatin in breast cancer to contribute to cell and tumor fitness and highlight how epigenetic activity may inform the therapeutic response. We conclude by providing perspectives on the future of therapeutic targeting of epigenetic enzymes, an approach that holds untapped potential to improve breast cancer patient outcomes.

## 1. Introduction

### 1.1. Breast Cancer Classification

A high degree of breast cancer heterogeneity led to its classification into four distinct molecular subtypes, which can vary in tumor genomics and the cell type from which tumor initiation occurs: (1) luminal A, (2) luminal B, (3) human epidermal growth factor receptor 2 (HER2)-positive, and (4) triple-negative breast cancer (TNBC). Tumor heterogeneity informs patient prognosis and clinical disease management. At diagnosis, a combination of immunohistochemistry (IHC) and genomic assays define critical gene expression of three cell surface receptors: estrogen receptor (ER), progesterone receptor (PR), and HER2. Collectively, ER, PR, and HER2 expression enable breast cancer classification into one of the four established molecular subtypes, which forms the basis of clinical disease management. Approximately 60–70% of all diagnosed breast cancers fall under the luminal A subtype, which is defined as ER-positive and/or PR-positive, HER2-negative, with low proliferative capacity by Ki67 IHC [1]. These tumors often respond to hormone therapies, including tamoxifen or aromatase inhibitors (AIs), resulting in a favorable patient prognosis [2,3]. In contrast with luminal A, luminal B breast cancers are more aggressive with a high proliferative index via Ki67 IHC. Luminal B breast cancers represent about 10% of breast cancers; these tumors are ER-positive and exhibit either PR-positive or PR-negative staining [2]. Approximately 30% of diagnosed luminal B cancers are also HER2-positive [4]. Patients with luminal B breast cancers do not exhibit as high a response rate to hormone therapy as patients with luminal A breast cancers, and some patients benefit from hormone therapy in conjunction with chemotherapy [5]. About 10–15% of diagnosed breast cancers are characterized by high HER2 expression in the absence of ER or PR expression [3]. HER2-positive patient prognosis has dramatically improved in the past 30 years with the development and widespread clinical application of HER2-targeted therapies, including small molecule inhibitors (e.g., lapatinib) and antibody therapies (e.g., trastuzumab), in combination with chemotherapies such as doxorubicin [6]. HER2-targeted antibody-drug conjugates such as trastuzumab-emtansine (T-DM1) or trastuzumab-deruxtecan (T-Dxd) are also used clinically for HER2-positive breast cancers as well as advanced breast cancers characterized by low HER2 levels [7,8]. Lastly, TNBCs account for 10–20% of diagnosed breast cancers, typically originate from the basal cell lineage in the breast, and are not characterized by ER, PR, or HER2 expression [9]. Instead, TNBC tumor genomics varies, resulting in proposed subclassifications within the TNBC subtype, and can be characterized by loss-of-function mutations in tumor suppressors such as *BRCA1/2* or *TP53* [10]. The heterogeneity of TNBCs and their aggressive nature render this tumor type more challenging to clinically manage, and patient prognosis is usually poor.

In addition to ER, PR, and HER2 expressions that stratify breast cancers into the above molecular subtypes, additional genomic alterations may be present that contribute to disease etiology, therapeutic response, and disease progression. This involves genomic alterations, including mutations and indels in *BRCA1/2*; inactivating mutations or deletions in tumor suppressor genes, such as *PTEN*, *CDH1*, or *TP53*; and amplification or activation of mutations in critical growth regulators, such as *PIK3CA* [11,12,13,14]. While the importance of genomic alterations in breast cancer clinical management cannot be understated, in recent years it has become clear that the epigenetic landscape provides an additional and dynamic layer of insight into the dysregulation of cellular processes in breast and other cancers. In some cases, tumor genomics and epigenetics are linked; genomic alterations have been defined as modulating the activity or function of chromatin-modifying enzymes, in turn playing a role in epigenomic regulation [12,15]. Posttranslational epigenetic modifications that occur on histones and on DNA can alter chromatin accessibility by modifying histone–DNA interactions, recruiting transcriptional machinery to DNA, or interacting with transcription factors [16]. Delineating the specific mechanisms by which these modifications fine-tune cellular processes in normal and cancerous conditions first necessitates an examination of their environment in the structure of chromatin.

### 1.2. Nucleosome Organization and Modification Inform Gene Expression

The size of the human genome in relation to the volume of the nucleus presents a need to organize the genome into a more condensed form. Histone hetero-octamers containing two of each of the core histone proteins—H2A, H2B, H3, and H4—organize DNA through compaction, forming nucleosomes [17,18]. The nucleosome is incorporated into chromatin through the winding of DNA, such that 145–147 base pairs of DNA are wrapped around each nucleosome. Histone proteins contain a globular core domain and unstructured N- and C-terminal tail domains, which extend out of the globular domain on either side [17,19]. The nucleosome is assembled through interactions within the globular domains of histone proteins, and together with nucleosome-incorporated DNA, the histone globular and tail domains support post-translational modification (PTM), which can inform transcriptional competence [20]. Covalent histone PTMs are orchestrated through the dynamic activity of chromatin-modifying enzymes: writers that introduce modifications, readers that identify and bind to PTMs, and erasers that remove the modifications [21]. Histones harbor more than 20 types of unique epigenetic PTMs, including methylation, acetylation, phosphorylation, SUMOylation, ADP ribosylation, and numerous others [22,23]. Collectively, the specific combination of histone PTMs can impact chromatin accessibility and stability, thereby regulating transcription and gene expression [24]. Beyond transcriptional regulation, the dynamic histone PTM landscape informs diverse cellular processes ranging from DNA repair to cellular differentiation [25,26]. Changes to the epigenome in the form of dysregulated PTMs contribute to cancer while also informing therapeutic responses and clinical patient outcomes. Collectively, discoveries in this research domain have led to the recognition of epigenetic reprogramming as an emerging, distinct hallmark of cancer [27].

Here, we examine the contributions of histone PTMs and the associated epigenetic modifiers that read, write, and erase these PTMs to the breast cancer landscape. DNA methyltransferases catalyze the methylation of cytosine residues to suppress transcription of the methylated gene. Their associated DNA methylation events are established regulators of breast cancer pathogenesis and clinical outcome and are reviewed extensively elsewhere [28,29,30,31]. As such, here we focus on preclinical mechanistic studies demonstrating the role(s) of histone-associated epigenetic modifiers in essential biological processes that inform cancer initiation, development, and therapeutic response. We conclude by examining how these mechanistic studies could potentially support the clinical management of breast cancer.

## 2. Histone-Modifying Complexes

### 2.1. COMPASS Complex Perturbation and Associated Histone Methylation in Breast Cancer

The activity of multiprotein, H3K4 methyltransferase-specific COMPASS (complex of proteins associated with Set1) complexes is dysregulated in breast cancers. A total of six Set1/MLL methyltransferases (Set1A-B, MLL1-4) recruit unique and shared subunits to support COMPASS complex substrate specificity across genome-wide mono-, di-, and tri-methylation of H3K4 [32]. H3K4 methylation is typically associated with transcriptional activation. In HR-positive, *PIK3CA* mutant breast cancer, clinical PI3K inhibition hyperactivates ER signaling. Mechanistic studies have found that the AGC kinase AKT phosphorylates the MLL4/KMT2D methyltransferase, reducing its methyltransferase activity; PI3K inhibition enhances MLL4 activity, increasing enhancer H3K4me1 and promoting an open chromatin state [33]. Functionally open chromatin supports the recruitment of the FOXA1 and PBX1 pioneer transcription factors to the genome to support ESR1 binding and subsequent transcription of ER-responsive genes. Other AGC kinases, including SGK1, mediate MLL4/KMT2D phosphorylation [34], suggesting that a wider network of growth factor-associated signal transduction pathways may integrate cellular cues with chromatin-modifying enzyme function to regulate gene expression. The importance of maintaining appropriate H3K4 methylation is highlighted by a report that demonstrates the H3K4me2/3 demethylase KDM5A is also an AKT effector. AKT-driven KDM5A phosphorylation increases KDM5A cytoplasmic localization, thereby decreasing KDM5A association with chromatin and increasing promoter H3K4me3 [35]. An increase of promoter H3K4me3 occurs at cell cycle-regulated genes, supporting the expression of genes involved in cell cycle progression, which is correlated with a poor patient outcome in advanced-stage breast cancers across molecular subtypes, including HR-positive breast cancers [35]. Targeting the MLL1 and/or the MLL4/KMT2D COMPASS complexes to reduce promoter or enhancer H3K4 methylation, respectively, impairs HR-positive breast cancer cell and tumor growth [33,36].

### 2.2. Dysregulation of SWI/SNF in Advanced Stage Breast Cancers

The SWItch-mating type/Sucrose Non-Fermenting (SWI/SNF) family of ATP-dependent chromatin remodeling complexes regulate gene expression by supporting DNA accessibility, affecting cell proliferation, differentiation, and the cellular response to DNA damage [37]. The ATPases BRG1 (SMARCA4) and BRM1 are common to all SWI/SNF complexes (BAF, P-BAF, and ncBAF), with unique cofactors and binding partners assembling to form each of the three unique complexes. Genomic alterations in the SWI/SNF complexes are relatively rare in primary breast cancers. However, genomic alterations in the BAF complex tumor suppressor and DNA-binding protein ARID1A occur in 12% of metastatic breast cancers and contribute to SWI/SNF dysregulation in breast and other cancers. Specifically, ARID1A deletion or functional inactivation is associated with poor patient prognosis in metastatic luminal A and HER2-positive breast cancers, including endocrine therapy-resistant, ER-positive breast cancers [38]. Recent studies using genome-wide CRISPR screens demonstrate that genes encoding the BAF complex—including *ARID1A*—mediate therapeutic response to ER antagonists; loss of ARID1A enhances proliferative capacity, rendering ER-positive breast cancer cell lines resistant to both tamoxifen and fulvestrant [39]. ARID1A and BRG1 physically associate with ER. This interaction supports ARID1A repression of ER-dependent transcription; this occurs by ARID1A binding to chromatin at ER-regulated enhancers using a FOXA1-dependent mechanism [39]. Complementary studies suggest that ARID1A expression supports luminal breast lineage maintenance. ARID1A loss impairs SWI/SNF recruitment to regions of the genome that support luminal lineage commitment, thereby mediating a transition to basal-like breast cancer cells that are ER-independent [40]. These studies suggest mechanisms of resistance to ER antagonists and additional therapeutic strategies to treat advanced ER-positive breast cancers characterized by genomic alterations in BAF complex components. Outside of ER-positive breast cancers, SWI/SNF complex function may also be linked to other breast cancer subtypes. BRG1 increases cell proliferation via activation of cell cycle-dependent genes in TNBC cell lines [41], and genetic loss of BRG1 increases histone deacetylase 1 (HDAC1) binding to the genome [41]. The transcription factors SOX4 and BRG1 cooperate to positively regulate PI3K/AKT signaling via transcription of *TGFBR2* [42]. Because TNBCs are characterized by high BRG1 levels [43], these reports suggest a patient cohort whose tumors may be sensitive to BRG1 and/or PI3K inhibition.

## 3. Histone Acetyltransferases

**Figure 1:**
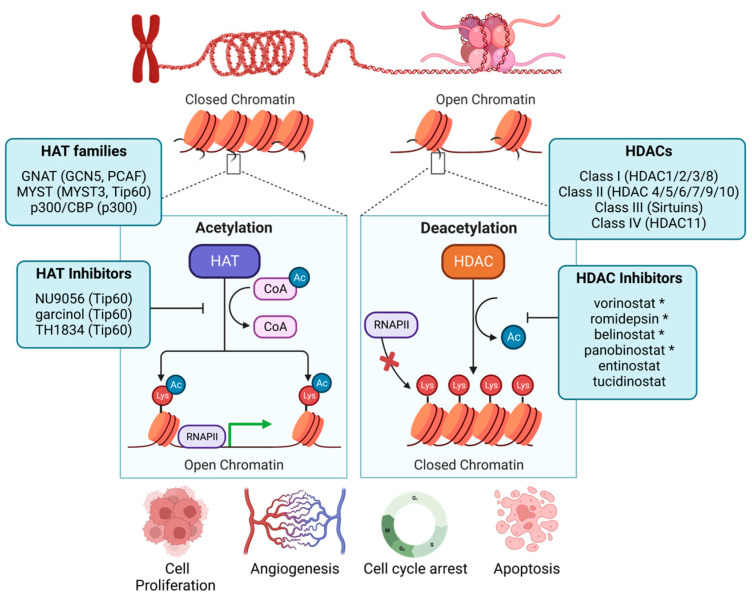
Epigenetic regulation of histone acetylation and deacetylation. HATs and HDACs have a crucial role in gene regulation. These enzymes are responsible for the transfer and removal of acetyl groups from lysine residues in histones. HDACs are classified into class I (HDAC 1, 2, 3, and 8), class II (HDAC 4, 5, 6, 7, 9, and 10), class III (SIRT1-7), and class IV (HDAC 11). HAT-directed lysine acetylation on histones promotes an open chromatin structure that enhances transcriptional competence. HDAC-directed lysine deacetylation supports chromatin compaction, thereby reducing transcriptional activity. Dysregulation of these chromatin-modifying enzymes aids in aberrant cellular proliferation, angiogenesis, epithelial-to-mesenchymal transition, and escape from cell cycle arrest and evasion of apoptosis. While HAT inhibition remains in preclinical development, four HDAC inhibitors are currently FDA-approved for human cancers and are denoted by *.

Histone acetylation is a reversible and dynamic PTM catalyzed by histone acetyltransferases (HATs) and removed by histone deacetylases (HDACs) [44]. HATs catalyze the covalent transfer of the acetyl group from the metabolite acetyl-CoA to lysine groups in proteins, including histones (Figure 1). While the activity of many HATs was originally described using histones as substrates, HATs have since been shown to acetylate several non-histone targets, including p53, YY1, and STAT3 [45,46,47]. Human HAT enzymes are divided into three major categories based on their sequence homology and mechanism of catalysis: the GNAT, MYST, and p300/CBP families (Figure 1) [48]. Functionally, HAT enzyme-mediated histone tail acetylation neutralizes the attraction between nucleosome-incorporated histone proteins and DNA, supporting a euchromatic state characterized by enhanced transcription [49,50]. A diverse array of acetylation events have been defined on histone H3 and H4 (including but not limited to H3K4 [51,52], H3K9 [53], H3K18 [54], H3K23 [55], H3K27 [56,57,58], H3K56 [59], H3K122 [60], H4K5 [61], H4K8 [62], H4K12 [62], and H4K16 [62]) [63], but their biological functions have not been well-characterized. For example, H3K27ac is widely recognized as a marker of active enhancers [64], but its specific mechanistic role in transcription is not completely understood. One method to contextualize these marks within breast cancer is to examine the enzymes that write or erase them and their catalytic functions within critical and commonly dysregulated cellular processes, including proliferation, differentiation, cell survival, and others.

### 3.1. Histone Acetylation in Breast Cancer Pathogenesis

While histone acetylation is critical for normal cellular function, histone acetylation is also subject to dysregulation in human cancers. Aberrant histone acetylation has been associated with cancer progression and therapeutic response [65,66,67,68]. This is highlighted by a clinical study examining a panel of histone acetylation marks in 880 human breast carcinomas. While hypoacetylation of H4K16 was observed in 78.9% of breast cancer cases, H4K16 hyperacetylation was correlated with longer disease-free survival (DFS), thus leading authors to postulate that loss of H4K16ac is an early event in breast cancer pathogenesis [65]. The presence of histone acetylation is also associated with different breast cancer subtypes. The overall detection of high levels of global histone acetylation was significantly associated with luminal-like breast tumors, whereas moderate to low global lysine acetylation was linked to basal carcinoma and HER2-positive tumors [65]. More specifically, an evaluation of several histone PTMs, including acetylation marks from patient tumors, suggested that H3K9ac is associated with TNBC and HER2-positive breast tumors, whereas H3K27me3 correlates with the luminal A and B molecular subtypes [69]. Furthermore, HAT-mediated acetylation has been shown to impact tumor phenotypes directly. MYST3 binds to the proximal promoter region of the *ESR1* gene, increasing ERα expression in a HAT-dependent manner; mutations in the HAT domain of MYST3 fail to upregulate ERα expression to the same extent as wild-type MYST3 [70]. Knockdown of MYST3 in HR-positive breast cancer xenografts leads to significant tumor regression and increased progression-free survival (PFS), corroborating in vitro studies that found that the genetic ablation of MYST3 significantly reduces cell growth in HR-positive breast cancer cell lines [70]. These data suggest that further exploration of MYST3-targeting for the treatment of MYST3-high ER-positive/HER2-negative breast cancers is warranted and may benefit endocrine therapy-resistant patients. While HATs catalyze the addition of acetyl groups to histone residues, the overall maintenance of histone acetylation consists of a dynamic interplay between HATs and HDACs, both of which can be dysregulated in breast and other cancers.

### 3.2. HATs Promote the Transcription of EMT-Specific Markers in Breast Cancer

HATs have been documented to function in the epithelial-to-mesenchymal transition (EMT). While EMT occurs normally during embryonic development, it has been implicated in the acquisition of stem-cell-like qualities that accentuate tumor growth and metastasis in cancer [71]. In noncancerous epithelial breast cells, DOT1L, a methyltransferase responsible for catalyzing the active transcription mark H3K79 methylation [72,73], has been shown to enhance the expression of EMT transcription factors in a p300-dependent manner. DOT1L recruits HAT p300 to the promoters of mesenchymal regulator genes *SNAI1*, *ZEB1*, and *ZEB2*, as part of a transcriptionally active complex containing DOT1L, c-MYC, and p300 [74]. This is concurrent with H3 acetylation enrichment at the *SNAI1, ZEB1*, and *ZEB2* promoters, alongside an increase in H3K79 methylation [74]. Additionally, in the TNBC cell line MDA-MB-231, pharmacologic HDAC inhibition with trichostatin A-induced re-expression of Snail, ZEB1, and ZEB2 transcription factors in DOT1L-knockdown cells reinforced the EMT and cancer stem cell-promoting function of p300 in the presence of DOT1L [74]. This study highlights how histone acetylation and its crosstalk with histone methylation marks can dictate tumor differentiation and subsequent aggressiveness. Separately, elevated activity of the GNAT family HAT GCN5 supports EMT by inducing the expression of Snail and Slug upon TGF-β1 treatment in TNBC cell lines, including MDA-MB-231 [75]. Functionally, this results in enhanced cell migration and invasion, which could facilitate cancer metastasis. Genetic knockdown of GCN5 reverses the metastatic phenotypes and prevents EMT [75]. Thus, the epigenome and its HAT-mediated regulation may contribute to phenotypes that support breast cancer metastasis (Figure 1).

### 3.3. HAT-Mediated Regulation of the DNA Damage Response

The HAT p300/CREB-binding protein-associated factor (PCAF) acetylates H4K8 in BRCA-deficient TNBC, thereby promoting replication fork degradation [76]. This study suggests that PARP inhibitor resistance in BRCA-deficient breast cancer may be due in part to low PCAF levels; PCAF levels could serve as an indicator to assess therapeutic response to PARP inhibition in BRCA-deficient breast and other cancers. These data simultaneously reinforce other studies that identified drug synergy via the combined use of HDAC and PARP inhibitors in various cancer types [77,78,79,80] and predict that this combination therapy may increase fork degradation and subsequent cell death in BRCA-deficient cancers [76]. Similarly, Tip60, a HAT in the MYST family, regulates homologous recombination-directed DNA repair in both normal and tumor mouse mammary epithelial cells (MECs) [81]. Genetic loss of Tip60 elevated MEC γH2AX [81], a marker for DNA damage. Surprisingly, Tip60-silenced MEC tumor cells were also markedly more resistant to the DNA-damaging agent cisplatin, despite an increase in unrepaired DNA compared to vehicle-treated cells [81], suggesting a dual role of Tip60 in both DNA damage repair and DNA damage-induced cell death. As such, HAT inhibitors are in preclinical development, with early studies suggesting activity in preclinical breast cancer models. The Tip60 inhibitor TH1834 induces apoptosis by generating non-repairable DNA damage and elevated H4K8ac [82] in HR-positive MCF7 breast cancer cells and slows xenograft growth in vivo [83]. Another Tip60 inhibitor, garcinol, inhibits estradiol-induced cell proliferation by enhancing G0/G1 cell cycle arrest and increasing apoptosis in MCF7 cells [84]. Collectively, the development and preclinical testing of HAT inhibitors that target specific HATs in human cancers are in their infancy. To date, HAT inhibition has not entered clinical trials, at least in part because HATs serve both oncogenic and tumor-suppressive roles within the cell. Comparatively, modulating histone acetylation through the inhibition of HDAC activity in breast and other cancers has gained more preclinical and clinical traction.

## 4. Histone Deacetylases

HDACs oppose HAT activity by catalyzing the removal of acetyl groups from the ε-amino group of a lysine residue, thereby restoring its attraction to DNA (Figure 1) [85]. Similar to HATs, HDACs have been reported to deacetylate several non-histone proteins, including p53 and STAT3 [86]. Eighteen HDACs are encoded in the human genome, divided into four classes based on protein homology with budding yeast. Class I HDACs (HDACs 1, 2, 3, and 8) are homologous to the reduced potassium dependency 3 (Rpd3) protein in *S. cerevisiae* [87]. HDAC8 is the only class I HDAC that has been shown to function as a monomer; other class I HDACs are recruited to larger complexes, including CoREST, MiDAC, NuRD, Sin3A (HDACs 1/2), and SMRT/NCoR (HDAC3) [88,89]. Class II HDACs are genetically similar to the Hda1 yeast protein [90], stratified into class IIa (HDACs 4, 5, 7, and 9) and class IIb (HDACs 6 and 10) based on domain composition [87]. HDAC11 is the sole member of the class IV HDAC family and is homologous to budding yeast Hos3.1 [87]. Class III HDACs (SIRTs 1, 2, 3, 4, 5, 6, and 7) are homologous to yeast Sir2 and are NAD-dependent enzymes, differing from class I, II, and IV HDACs, which all perform zinc-dependent catalysis [87]. HDACs can be found in numerous cellular contexts and compartments; further defining their mechanistic roles in cancer may support future studies to target their oncogenic activity or exploit their potential prognostic value. Generally, class I and II HDACs have been shown to have oncogenic roles, while class III HDACs exhibit both oncogenic and tumor-suppressive activity [91]. The sole class IV HDAC11 can maintain tumor cell viability and metabolism while preventing apoptosis, supporting its role as an oncoprotein [92]. Given the described oncogenic activity of class I HDACs, therapeutic targeting of class I HDACs remains the most clinically advanced. Companion genetic studies demonstrate that individual genetic deletion of class I HDACs (HDAC1, 2, 3, or 8) does not reduce cancer cell viability, highlighting the redundant function of class I enzymes and supporting the utility of pan-HDAC inhibitors [93]. Haberland and colleagues also demonstrate that combined genetic deletion of HDAC1 and HDAC2 increases cell death and mitotic catastrophe, like pan-HDAC pharmacological inhibition [93]. These studies suggest that true pan-class I HDAC inhibition may not be necessary to exhibit anti-tumorigenic effects.

### 4.1. HDACs as Prognostic Factors in Breast Cancer Patients

In general, HDACs are overexpressed in breast and other cancers [94,95,96,97,98,99]. At the same time, the molecular mechanism(s) by which HDACs contribute to tumorigenesis are not yet fully understood. High HDAC1 expression is positively correlated with hormone receptor status in patient tumor samples [100,101,102]. HDAC1 and HDAC2 expression are significantly correlated with HER2 expression [101,102], and HDAC2 expression is associated with the presence of nodal metastasis [101]. Despite detectable HDAC overexpression in some breast cancer subtypes, the ability of HDAC overexpression to predict overall survival (OS) or DFS is less clear; conflicting results were published indicating the potential prognostic value of HDAC6 [102,103] and HDAC2 [101,102,104] expression. These discrepancies in the potential prognostic value of HDAC expression could, in part, be due to differences in race and ethnicity in participating patient populations, therapeutic regimens, post-operative treatment protocols, or tumor staging [102]. However, these reports also reinforce the notion that HDACs have diverse, tissue-specific roles that vary between the HDAC classes described above, and those specific functions, rather than an overall characterization of HDACs, could guide clinical approaches in the therapeutic targeting of HDACs. Therefore, it is critical to consider HDAC functions in specific tumorigenic contexts, which may vary widely by breast cancer subtype, tumor staging, or prior therapeutic regimens.

### 4.2. HDACs Support the Epithelial-to-Mesenchymal Transition (EMT) in Breast Cancer

The Nucleosome Remodeling and Deacetylation (NuRD) complex is a repressor complex that reduces DNA accessibility within chromatin [105]. The complex contains class I HDAC1 and HDAC2, an ATP-dependent chromatin remodeling protein (CHD3/4/5), methyl-CpG-binding domain protein (MBD2/3), nuclear zinc finger protein (Gata2a/2b), SANT and GATA DNA-binding domains (MTA1/2/3), as well as additional scaffolding proteins (Rbbp4, Rbbp7) [105]. Prior studies identified that NuRD interaction with the EMT-related transcription factor ZEB1 contributes to non-small cell lung cancer metastasis [106]. In TNBC, the transcription factor RUNX2 has been shown to recruit NuRD to repress the expression of tumor suppressor genes, thereby promoting EMT. RUNX2 binds with the NuRD complex member MTA1, and either RUNX2 or MTA1 overexpression augments fibronectin, N-cadherin, and vimentin mesenchymal markers while depleting E-cadherin, α-catenin, and γ-catenin epithelial markers [107]. The HDAC class I/II inhibitor vorinostat upregulates E-cadherin, enhancing differentiation in TNBC cells [108]. The authors found that class I/II HDAC inhibition via vorinostat or entinostat decreases protein expression of TCF4, a major Wnt signaling effector. In this context, HDAC inhibition reprograms the epigenome to repress Wnt signaling [108], thereby reducing EMT [109,110,111]. These data suggest that HDAC inhibition may support cancer cell differentiation, reducing tumorigenesis while improving treatment efficacy. In a murine mammary carcinoma model, *HDAC8* siRNA knockdown reverses the upregulation of EMT-driver genes *VIM*, *CDH2*, *WNT5A*, and *ZEB1* in surviving cells following treatment with cisplatin and/or paclitaxel [112]. Conversely, *HDAC8* knockdown rescued the chemotherapy-induced downregulation of gene transcripts whose protein products are associated with mesenchymal-to-epithelial transition (e.g., *ELF*, *GATA3*, *RORA*, and *GRHL2*), supporting the epithelial phenotype by reversing EMT [112]. These same genes exhibited a significant decrease in promoter-proximal H3K27ac occupancy following chemotherapy [112], suggesting HDAC8-mediated EMT antagonizes chemotherapy efficacy. HDAC inhibition via entinostat hinders EMT, reducing the expression of the EMT transcription factors Twist and Snail as well as N-cadherin [113]. Chromatin immunoprecipitation (ChIP) demonstrates that Twist and Snail binding to the E-cadherin-encoding *CDH1* promoter decreases with entinostat treatment, leading to an increase in *CDH1* mRNA expression [113]. This coincides with earlier studies that have found that Snail represses CDH1 by recruiting the Sin3A-HDAC1/2 complex to the *CDH1* promoter [114]. Cytokeratin 8/18, filaments lost during EMT [115], were upregulated via entinostat treatment [113]. Numerous HDACs have been implicated in the EMT process; inhibiting HDACs that regulate gene expression associated with EMT may enhance the anti-tumorigenic effects of other therapies, including DNA-damaging agents commonly used in breast cancer treatment.

### 4.3. HDACs Modulate ER Expression and Signaling

The NuRD complex may also repress ER expression in breast cancer. In advanced-stage TNBC, the NuRD complex interacts with the *ESR1* promoter, which reduces promoter H3K27ac via an HDAC1-dependent mechanism, thereby reducing ERα expression [116]. Further, when NuRD is suppressed by knocking down the NuRD-recruiting protein MUC1, an increase in *ESR1* promoter H3K27ac is observed, along with induction of ERα expression at both mRNA and protein levels [116]. Complementary studies in melanoma found that the NuRD complex is recruited to the Twist-binding chromatin region of the *ESR1* gene by Twist to decrease H3K9ac and increase H3K9me1 in melanoma, consistent with the repressive role of NuRD in ERα expression [117]. These results suggest that at least some HDACs are involved in epigenetic remodeling, which may support the transition of luminal breast cancer into more aggressive basal breast cancer. Pharmacologically targeting HDACs that contribute to NuRD complex function may reverse the de-differentiation of TNBC and enhance ER expression, which could potentially define new patient populations who may benefit from hormone therapy.

NuRD complex constituents also play a role in ER pathway signaling. Within the normal estrogen pathway, estrogen binds to estrogen-responsive elements (EREs) to transactivate downstream genes [118]. The NuRD complex member MTA1 suppresses ERE-mediated transcription by recruiting HDACs and interacting with the activation domain of ERα in HR-positive breast cancer [119]. Class I HDAC inhibition via trichostatin A mediates a reversal of the inhibition of ERE transcription by MTA1 [119]. HDAC2 localizes with MTA1 to the *PS2* promoter, which is an ER-responsive gene and a tumor suppressor [119,120]. Other studies in HR-positive breast cancer demonstrate that HDAC1 and HDAC3 are recruited to the *PS2* and *c-MYC* promoters in a tamoxifen-dependent manner as part of chromatin-remodeling complexes: HDAC3-containing NCoR and HDAC1-containing NuRD [121]. The introduction of tamoxifen reverses estrogen-induced H3 and H4 acetylation, leading to global H3 hypoacetylation and an increase in heterochromatin, as well as a reduction in RNA pol II occupancy at *PS2* and *c-MYC* genes [121]. The ER antagonist tamoxifen is one of the most common first-line treatment drugs for patients with ER-positive breast cancer [122,123]. However, tamoxifen resistance occurs in 50% of these tumors [123]. Targeting HDACs may reduce tamoxifen resistance, which could restore hormone therapy benefits in some patients with HR-positive breast cancers.

### 4.4. HDACs as a Therapeutic Target to Overcome Treatment Resistance

The upregulation and nuclear localization of the embryonic transcription factor SOX9 support tamoxifen resistance in ER-positive breast cancer [124]. SOX9 upregulation is sufficient to cause resistance to estrogen deprivation, along with a decreased sensitivity to tamoxifen [124]. This may, in part, be due to the ability of SOX9 to increase survival and confer metastatic properties in cancer [125,126]. HDAC5-mediated SOX9 deacetylation induces SOX9 nuclear localization in HR-positive breast cancer, and HDAC5 overexpression promotes cell growth under tamoxifen exposure [127]. Notably, c-MYC regulates the transcription of *HDAC5* [127]. C-MYC maintains the nuclear localization of SOX9 in an HDAC5-dependent manner; HDAC5 overexpression rescues a reduced growth rate in tamoxifen-resistant MCF7 cells that have been treated with shRNAs targeting *c-MYC* [127].

Radiotherapy remains a treatment mainstay for many breast cancer patients. However, radiation resistance occurs through many mechanisms, including intra-tumoral heterogeneity or activation of downstream signaling pathways [128], such as ERK1/2 and PI3K/AKT [129]. Several studies suggest that HDACs may also contribute to radioresistance in other cancers [130,131,132,133]. Thus, HDAC inhibition in breast cancer may enhance radiotherapy efficacy, improving patient outcomes. To investigate the mechanisms of radioresistance, radioresistant HR-positive and TNBC cell lines have been engineered and are characterized by reduced H3K9ac and H3K27ac and an increase in HDAC activity compared to radiosensitive parental cell lines [134]. In radioresistant HR-positive breast cancer cell lines, the loss of H3K9ac and H3K27ac occurred independently of the cell cycle, suggesting that these events are connected to the acquisition of radioresistance [134]. Treatment with the class I HDAC inhibitor valproic acid increases H3K9ac and H3K56ac while also increasing γH2AX retention, signaling sustained DNA damage, in both acquired and intrinsically radioresistant cells [134]. These studies suggest that HDAC inhibition may sensitize some breast cancer subtypes to DNA-damaging agents, including radiotherapy, although a more thorough investigation is necessary to determine the broader implications of these studies.

### 4.5. Clinical HDAC Inhibition

**Figure 2:**
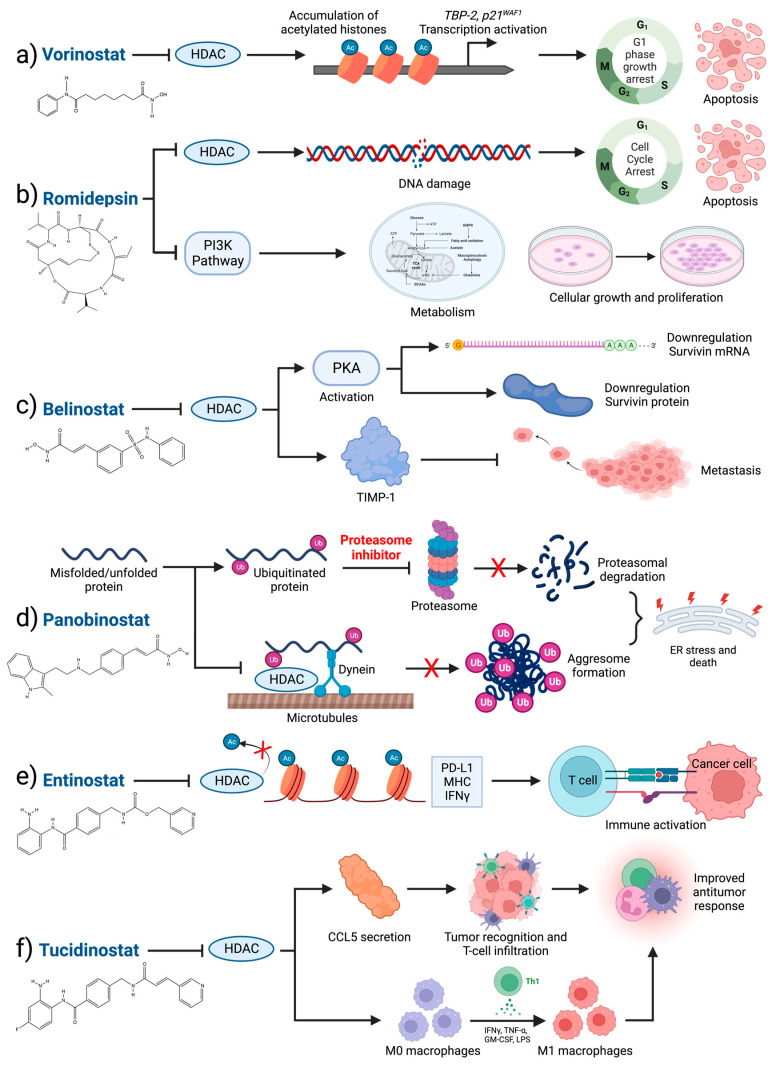
Functional mechanisms of the clinically viable HDAC inhibitors. (**a**) Vorinostat inhibits HDAC activity, resulting in the accumulation of hyperacetylated histones H3 and H4, leading to the transcriptional activation of genes including *TBP-2* and *p21^WAF1^* [161,162]. The induced activation of *p21^WAF1^* supports G1 cell cycle arrest followed by apoptosis. (**b**) Romidepsin inhibits cellular proliferation via the accumulation of hyperacetylated histones, resulting in replication fork delays and DNA double-strand break formation [163]. Thus, romidepsin-driven DNA damage induces cell cycle arrest and apoptosis. Romidepsin also inhibits the PI3K pathway, a master regulator of cell metabolism, growth, proliferation, and survival [164]. (**c**) HDAC inhibition via belinostat leads to PKA-dependent downregulation and degradation of survivin, mediating cell death [165]. Additionally, belinostat induces TIMP-1 expression, which may decrease tumor cell invasion and inhibit metastasis [166]. (**d**) The HDAC inhibitor panobinostat synergizes with the proteasome inhibitor bortezomib to block the proteasomal degradation and aggresome accumulation of ubiquitinated misfolded proteins. Ubiquitinated proteins are normally transported via HDAC6 association and the dynein motor for aggresome processing. HDAC inhibition via panobinostat results in the accumulation of misfolded proteins, inducing endoplasmic reticulum (ER) stress, and ultimately, cell death [167,168]. (**e**) Entinostat activates the transcription of immune response genes such as *IFNG*, *CD274 (*PD-L1), and genes encoding MHC proteins, which support immune activation and increase antigen presentation on tumor cells [169,170]. (**f**) Tucidinostat increases T cell-attracting chemokines, including CCL5, enhancing T cell intra-tumoral infiltration. Tucidinostat also modulates the M1 polarization of macrophages in solid tumors [171].

The pro-oncogenic activities of HDACs, along with structural and activity features that render HDACs viable therapeutic targets, provide sufficient rationale to develop and utilize HDAC inhibition for the clinical management of human cancers, including breast cancer [135]. To date, four HDAC inhibitors have achieved US FDA approval to treat patients with cancer (Table 1, Figure 1). In 2006, the class I/II HDAC inhibitor vorinostat was approved to treat patients with advanced cutaneous T-cell lymphoma (CTCL), which was followed by the 2009 approval of romidepsin for the same indication [136,137]. Romidepsin achieved accelerated approval as a class I HDAC inhibitor for peripheral T-cell lymphoma (PTCL) initially in 2011; however, its PTCL approval was withdrawn in 2021 due to its failure to reach the endpoint of PFS in combination with the chemotherapy CHOP [138]. The pan HDAC inhibitor belinostat was FDA-approved for the treatment of PTCL in 2014 [139], and the pan HDAC inhibitor panobinostat gained accelerated approval in 2015 to treat multiple myeloma in combination with dexamethasone and the proteasome inhibitor bortezomib [140]. Currently, the clinical utility of HDAC inhibition is restricted to the hematological malignancies described above. For solid tumors, HDAC inhibition remains under limited clinical investigation, with no therapies having advanced beyond stage III clinical trials for breast cancer (Table 1). While many mechanisms of action have been reported for HDAC inhibitors, including triggering DNA damage, inducing ER stress, and enhancing tumor-infiltrating lymphocytes (see Figure 2 for a selection), undiscovered mechanisms likely exist, given the functional diversity of HDAC enzymes. Due to the importance of HDACs and histone acetylation in breast cancer development and the metastatic cascade, the rationale remains for the continued exploration of clinical HDAC inhibition as part of a combination treatment regimen.

Currently, while preclinical mechanistic data support the utility of HDAC inhibition in multiple breast cancer subtypes via the unique mechanisms described above and elsewhere, HDAC inhibition is not an approved therapeutic modality for the treatment of breast cancer. Preclinical data demonstrated that the class I and II HDAC inhibitor entinostat significantly reduces the growth of letrozole-resistant tumors in the presence of AI letrozole or exemestane, overcoming AI resistance [141]. These data led to a US multi-center phase III clinical trial in patients with advanced HR-positive breast cancer, but the trial did not meet the endpoint in AI-resistant HR-positive/HER2-negative breast cancer when treated with exemestane plus entinostat [142]. Patients treated with exemestane plus entinostat showed a median OS of 23.4 months (95% CI, 21.2 to 25.6) compared to 21.7 months (95% CI, 19.3 to 27.1) for patients treated with exemestane plus a placebo (*p* = 0.94) [142]. Similarly, median PFS was 3.3 months (95% CI, 3.1 to 5.3) in patients treated with exemestane plus entinostat and 3.1 months (95% CI, 3.0 to 3.3) in the exemestane/placebo group (*p* = 0.30) [142]. This contrasts with a similar international study based in China that examined the use of the class I/II HDAC inhibitor tucidinostat with exemestane in the treatment of advanced, HR-positive breast cancer. Patients who received tucidinostat plus exemestane experienced 9.2 months of PFS compared to the placebo group’s 3.8 months (*p* = 0.024) [143]. This study was limited to a single racial patient cohort, and quality of life metrics were not reported. Furthermore, patients in this study were less likely to have received prior endocrine therapy for advanced disease, which could account for differences in efficacy between these two reported HDAC inhibitor trials [142,143]. Given the importance of HDACs in mediating multiple cancer hallmarks and the promising results of the above tucidinostat trial, preclinical and clinical HDAC inhibition should continue to be explored. Moving forward, rational combination therapies may enhance the on-target activity of HDAC inhibition.

**Table 1:** Clinically viable HDAC inhibitors and their targets. HDAC inhibitors that have achieved FDA approval in non-breast cancers or have an active, ongoing clinical trial in breast cancer. HDAC inhibitor classification, the HDAC inhibitor targets, the known histone acetylation marks that are regulated via the inhibitor, current approval status, existing clinical trials, and associated adverse events are indicated. When known, the context in which histone acetylation is regulated is described, along with the disease indication. While multiple HDAC inhibitors have entered US-based clinical trials for the treatment of breast cancer, 12 clinical trials are currently active.

Agent	Classification	Target(s)	PTM-Regulation	Approval Indication	Trial Stage in Breast Cancer	Clinical Trial Status	Reported Adverse Events ≥Grade 3
** Vorinostat (SAHA) **	Hydroxamic acid	HDAC1, 2, 3, 8 (Class I) and HDAC6 (Class IIb) [144]	H3K14ac and H3K27ac (MCF7); H3K27ac, H3K18ac, H4K5ac (MDA-MB-231) [145]; H3K9ac (TNBC) [146]	Cutaneous T-cell lymphoma (FDA) [147]	I, II	Active; NCT03742245, NCT00616967, NCT04190056, NCT03878524	Thrombocytopenia, Anemia, Deep vein thrombosis, Dehydration, Pyrexia, Hypotension, Pulmonary embolism, Sepsis [148], Nausea, Fatigue, Vomiting, Asthenia, Constipation, Hypokalemia [149]
** Romidepsin (FK228) **	Cyclic peptide	HDAC1, 2, 3, 8 (Class I) [150]	Unknown	Cutaneous T-cell lymphoma (FDA) [137]	I, II	Active; NCT02393794, NCT01638533	Anemia, Leucopenia, Neutropenia, Thrombocytopenia, Cardiac disorders, Eye disorders, Gastrointestinal disorders, General disorders and administration site conditions, Immune system disorders, Infections and infest, Metabolism and nutrition disorders, Nervous system disorders, Respiratory, thoracic, and mediastinal disorders, Skin and subcutaneous tissue disorders [151]
** Belinostat (PXD101) **	Hydroxamic acid	Pan-inhibitor for zinc-dependent HDAC [152]	Unknown	Relapsed/Refractory peripheral T-cell lymphoma (FDA) [139]	I	Active; NCT04315233, NCT04703920	Hypertriglyceridemia, Hemoglobin, Dyspnea, Fatigue, Dehydration, Hypoxia, Nausea, Vomiting, QTc prolonged, Dizziness, Hypercholesterolemia, Allergic reaction, Rash, Diarrhea, Tracheal hemorrhage, Left ventricular dysfunction, Small Bowel Obstruction, Palmar-plantar syndrome [153]
** Panobinostat (LBH-589) **	Hydroxamic acid	Pan-inhibitor for zinc-dependent HDAC [144]	H3K9ac, H4K8ac (TNBC) [154]	Multiple myeloma (FDA) [140]	I	Active; NCT03878524	Neutropenia, Thrombocytopenia, Diarrhea, Nausea, Infection, Upper Respiratory Tract Infection, Vomiting, Lower Respiratory Tract Infection [155]
** Entinostat (MS-275) **	Benzamide	HDAC1, 2, 3 (Class I), and HDAC9 (Class II) [156]	Unknown	Breakthrough designation (advanced breast cancer)	III	Active; NCT01349959, NCT02115282, NCT02453620, NCT03280563. NCT03538171 (China)	Anorexia, Nausea, Vomiting, Fatigue. Diarrhea, Leukopenia, Neutropenia, Thrombocytopenia, Hypoalbuminemia, Hypocalcemia, Hyponatremia, Hypophosphatemia, ALT [157]
** Tucidinostat **	Benzamide	HDAC1, 2, 3 (Class I), HDAC10 (Class II) [156]	H3K9ac, H3K18ac (TNBC, HR+) [158]	Peripheral T-cell lymphoma (CFDA)	III (China)	Active (China only); NCT05276713, NCT05390476, NCT05632848, NCT05633914, NCT05335473, NCT05411380, NCT04192903, NCT05747313, NCT05186545, NCT05047848, NCT05890287, NCT05749575, NCT05085626, NCT05464173, NCT05400993	Anemia, Leukopenia, Neutropenia, Thrombocytopenia, Increased alanine aminotransferase, Increased aspartate aminotransferase, Increased transpeptidase, Hypokalemia [159], Diarrhea, Lymphopenia, Decreased Appetite, Blood Alkaline Phosphatase Increased, Gamma-glutamyl Transferase Increased, Weight Decrease [160]

### 4.6. Perspectives on HDAC Inhibition in Breast Cancer

As described above and elsewhere, class I HDACs, including HDAC1/2/3, participate in repressive multi-unit complexes that have been shown to decrease ER expression and estrogen signaling, such as reducing pS2 and c-MYC expression. Moving forward, it may be valuable to investigate the action of class I-specific HDAC inhibitors for the treatment of advanced-stage breast cancer. Because of the role of class I HDACs in repressing ER expression and promoting EMT, there may also be value in the preclinical investigation of sequential class I HDAC inhibition followed by aromatase inhibition or selective estrogen receptor modulator (SERM) treatment in TNBC or other HR-negative breast cancers. We hypothesize that this approach could support TNBC de-differentiation into an ER-dependent tumor, which is therapeutically actionable. These proposed studies could expand on the clinical trial that utilized entinostat with exemestane, which focused only on overcoming aromatase inhibitor resistance in HR-positive breast cancers. Because HDAC5 has been shown to mediate tamoxifen resistance in HR-positive breast cancer [127], it may be worth developing and testing class II HDAC inhibitors for overcoming resistance in advanced-stage HR-positive breast cancer. Moreover, the class I/II HDAC inhibitors vorinostat and entinostat demonstrate preclinical efficacy in repressing EMT in TNBC cell culture models; testing vorinostat or entinostat in in vivo models of TNBC in combination with chemotherapy may demonstrate greater treatment efficacy, as it has been shown in other cancers that chemo-sensitivity significantly increases with tumor differentiation [172,173]. Lastly, the relationship and functional overlap between epigenetic and genetic mechanisms of tumor development and metastasis should be considered to maximize clinical benefit for breast cancer patients. Inhibitors such as the dual PI3K/HDAC inhibitor fimepinostat may overcome PI3K inhibitor resistance, given the established antagonism between PI3K and ER signaling. PI3K inhibition is associated with a compensatory increase in ER-dependent transcription [34,174], and HDAC inhibition has been shown to decrease ER-dependent transcription [119].

## 5. Conclusions and Perspectives

Despite the categorization of existing breast cancers into luminal A, luminal B, HER2, and triple-negative subtypes, intra-tumoral epigenetic heterogeneity and epigenetic plasticity can impact breast cancer aggressiveness and response to existing therapeutics. Targeting the enzymes and enzyme complexes responsible for these epigenetic changes may reduce breast cancer growth and proliferation. This epigenetics-forward approach may address some of the mechanisms of resistance to standard-of-care therapies commonly employed for breast cancer clinical management and identify mechanisms to promote tumor differentiation, which can open avenues for enhanced efficacy in later-line treatments. As more information is obtained about how the cellular roles of histone methyltransferases, HATs, HDACs, and chromatin-remodeling complexes are perturbed in cancer initiation, development, and therapeutic response, it remains critical to investigate their therapeutic targeting to advance current, personalized treatment strategies against breast cancer.
